# A digitally enabled home-based wound care program using the PEDALs model: a mixed-methods study protocol

**DOI:** 10.3389/fpubh.2025.1591187

**Published:** 2025-06-06

**Authors:** Lili Chen, Xuefang Zhuo, Hong Cai, Penghua Zeng, Hongyu Chen, Gang Chen

**Affiliations:** ^1^Shengli Clinical College of Fujian Medical University, Fuzhou, China; ^2^Department of Nursing, Fujian Provincial Hospital, Fuzhou, China; ^3^Fujian Key Laboratory of Geriatrics Diseases, Fujian Provincial Center for Geriatrics, Fuzhou, China; ^4^Department of Plastic and Burn Surgery, Fujian Provincial Hospital, Fuzhou, China; ^5^The School of Nursing, Fujian Medical University, Fuzhou, China; ^6^Department of Endocrinology, Fujian Provincial Hospital, Fuzhou, China

**Keywords:** chronic wounds, wound care, PEDALs model, digital health, home-based, telehealth, home care services, implementation research

## Abstract

**Background:**

Chronic wounds impose a significant burden on patients and caregivers because of complications such as pain, infection, and repeated trips to and from hospitals. These challenges are compounded by prolonged healing times and substantial socioeconomic costs, particularly in aging populations and resource-limited settings. The Digitally Enabled Home-Based Wound Care (DE-HBWC) program facilitates the organization of home visits by caregivers through electronic devices and internet platforms, enabling patients to easily receive medication changes and treatments, thus allowing patients to continue to reside in familiar surroundings while enjoying the same treatment as they would in a hospital. The construction of DE-HBWC programs for chronic wound patients in China is hampered by a lack of scientific theoretical guidance and high-quality research. The PEDALs (Problem, Evidence, Determinants, Actions, Long-term, Scales) model, a theoretical framework used in implementation research, as a rational framework for the DE-HBWC program in this study. Unlike conventional approaches, the PEDALs model emphasizes iterative adaptation and long-term scalability, ensuring alignment with real-world clinical needs and technological advancements.

**Methods:**

We designed a combined quantitative and qualitative study consisting of five phases and one monitoring step, following the PEDALs model. In Phase I, the clinical problem was defined as wound management challenges in patients with chronic wounds. In Phase II, a literature review and evidence-based practice were undertaken to address the research questions. In Phase III, in-depth interviews will be conducted to identify barriers and facilitators to the implementation of the DE-HBWC program in chronic wound patients. In Phase IV, the DE-HBWC intervention protocol will be developed and followed by a randomized controlled trial of the DE-HBWC intervention program. In Phase V, a DE-HBWC implementation plan that is sustainable over the long term will be developed to promote its use. Monitoring of the implementation of interventions in phases III to V will be conducted.

**Discussion:**

This study evaluated the impact of the DE-HBWC program on wound healing and quality of life in chronic wound patients and to assess its economic benefits. The findings are expected to help resolve challenges faced by patients with chronic wounds attending hospitals for wound care.

**Trial registration:**

Prospectively registered at the Chinese Clinical Trials Registry on 24.12.2023, ChiCTR2300079030.

## Introduction

Skin diseases can cause severe non-fatal disabilities worldwide, especially in resource-scarce areas, affecting nearly one-third of the world’s population (1, 2). Increasing research on skin diseases, with the aim of providing high-quality care, is crucial for alleviating the burden of skin diseases (2). Skin and subcutaneous diseases are the fourth leading cause of non-fatal disease burden, accounting for 1.79% of the global disease burden (1). Chronic wounds are wounds that have not healed or shown no signs of healing for more than 1 month because of various factors; such wounds include vascular ulcers, diabetes foot ulcers, pressure ulcers, and chronic infectious wounds (3). The incidence rate of chronic wounds caused by various etiologies in the general population is 2.21‰, with leg ulcers having the highest incidence rate of about 1.51‰ and chronic wound patients increasing at a rate of 10% per year (4, 5). Chronic wounds mainly occur in the older adult population, and are associated with a series of complications and medical expenses (6). Chronic wounds cost over 25 billion US dollars in medical expenses annually, with 5% of the total medical expenses used for wound repair (3). Owing to complications such as pain, infection, amputation, and repeated visits to hospitals for medical treatment, chronic wounds impose a significant burden on patients’ personal, family, economic, and social well-being (6, 7). It is expected that chronic wounds will continue to be a major clinical, social, and economic challenge owing to the aging population, continuing global threat of diabetes and obesity, and the persistent emergence of drug-resistant infections. Addressing public health issues related to chronic wounds requires multiple approaches, including prevention, improving wound care management, and addressing potential risk factors (8). In the long run, the development and expansion of remote home care for chronic wound patients may enable a larger patient population to be targeted and improve their compliance with treatment (9, 10).

Emerging digital health technologies, such as remote monitoring and artificial intelligence (AI)-driven wound imaging, hold transformative potential for decentralizing care (11). However, their integration into structured home-based programs remains underexplored, particularly in the context of chronic wound management (12). Research indicates that these technologies can yield significant economic and health benefits, including reduced infection risk, enhanced patient mobility, cost savings, and improved clinical outcomes (13–15). With advancements in mobile electronic facilities and internet technology, electronic-based home care has been successfully implemented for patients with various conditions, including chronic wounds (16, 17). The Digitally Enabled Home-Based Wound Care (DE-HBWC) program represents an innovative intervention that combines telehealth technology with home care approaches. It enables caregivers to conduct home visits via electronic devices and internet platforms, allowing patients to receive medications and treatments conveniently. This not only reduces hospitalization costs but also enables patients to remain in familiar surroundings while receiving comparable care to that provided in hospitals. However, in China, the DE-HBWC program for patients with chronic wounds is still in the initial stage of exploration, and further research is needed (18). The primary objectives of this approach are to alleviate the burden of hospital visits, improve continuity of care, and enhance treatment outcomes through real-time monitoring and data-informed decision-making.

Interventions in clinical nursing research are often controversial owing to the lack of scientific theoretical support. As an emerging discipline, implementation science is aimed at promoting the application of evidence-based intervention programs in practice. In recent years, such programs have received increasing attention in various research fields. Their implementation helps to transform theoretical possibilities into practical applications, thus addressing the challenge of implementing validated intervention measures in the real world (19, 20). In 2013, the World Health Organization recommended the application of implementation studies in the field of healthcare, clarifying which interventions are effective and the reasons for their success or failure during implementation, in order to ensure that interventions operate optimally in the real world (20). Some nursing scholars have also attempted to apply commonly used theoretical frameworks of implementation science to nursing research, narrowing the gap between knowledge and practice, confirming the practicality of interventions and optimizing valuable nursing resources. Nurses can also establish a solid evidence foundation for nursing clinical practice (21). The PEDALs model (22) is a new research theoretical framework in the field of implementation research. It was first proposed by Professor Xu Dong’s team and applied in diabetes sharing outpatient service and influenza vaccination programs. The basic steps of the PEDALs model are as follows: Phase I: P (Problem) is a real dilemma; Phase II: E (Evidence) refers to evidence-based practice for solving difficulties; Phase III: D (Determinants) is the determining factor for implementing evidence-based practice, including obstacles and promoting factors; Phase IV: A (Actions) are implementation strategies that promote evidence-based practices; Phase V: Long term (L), refers to a plan for continuous implementation; and Scale(s) is the monitoring and evaluation of the research process. The first letter of each of these steps gives rise to the acronym PEDALs. The PEDALs model was selected for its structured yet flexible approach to addressing implementation challenges in digital health. Unlike Consolidated framework for implementation research (CFIR) (23), which provides a static taxonomy of determinants, PEDALs integrates problem identification, evidence synthesis, and iterative adaptation into a cohesive workflow. This is critical in chronic wound care, where interventions must adapt to evolving patient needs and technological advancements. Few nursing-related studies based on the PEDALs model have been reported, both in China and other countries. Therefore, application of this theory to help nursing staff conduct implementation research more effectively in real-world situations, and to make the research process and results more scientific, would represent a novel step in this field.

Therefore, this study utilizes a mixed-methods approach to construct a digitally enabled home-based chronic wound care project for chronic wound patients based on the PEDALs model, and to evaluate the effectiveness of this project in chronic wound patients at home using a randomized controlled trial approach.

## Methods/design

### Design

**Figure 1:**
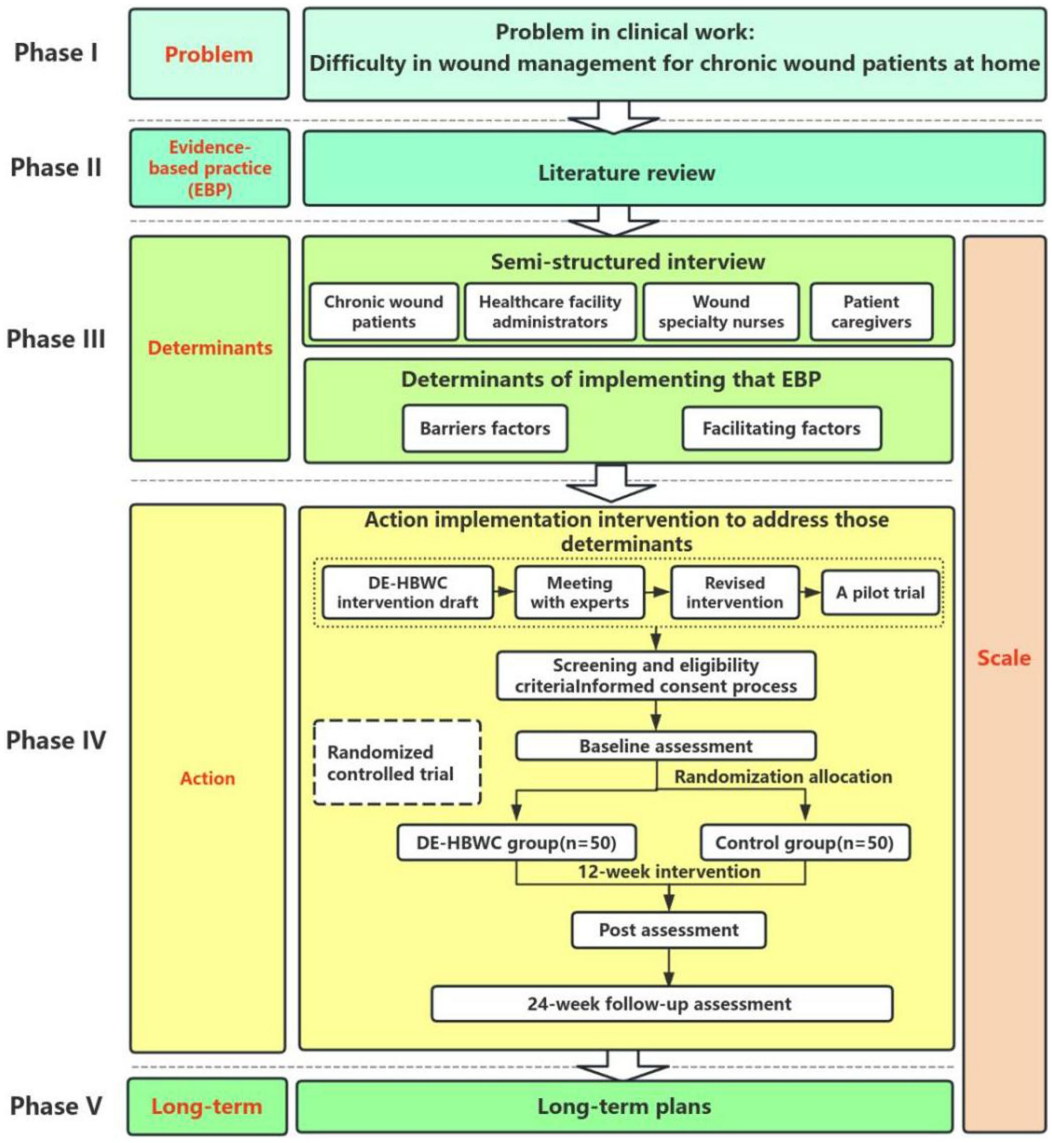
Flow diagram of the study design.

Our goal was to develop a DE-HBWC program for patients with chronic wounds, using scientific research methods. The study was a prospective study with a multi-stage mixed-methods design combining qualitative and quantitative research, following the five steps of the PEDALs model research paradigm (Figure 1). Qualitative methods included face-to-face one-on-one semi-structured interviews, literature review, and meetings with experts. The quantitative method comprised a parallel, two-armed randomized controlled trial design, with a 1:1 allocation ratio between the test and control groups for allocation concealment as well as blinding of assessors and statistical analysts.

### Setting

This study will be conducted at Fujian Provincial Hospital, Fuzhou City, Fujian Province, China, which is one of the largest tertiary hospitals in Fujian Province, with highly developed facilities, platforms and staff support.

### Methods

This study followed the five steps of the research paradigm of the implementation science PEDALs model.

#### Phase I: Problem

Based on their clinical work, wound clinic healthcare professionals have identified many real-life challenges facing homebound chronic wound patients, such as long wound treatment cycles requiring frequent trips to the hospital, inconvenient access to medical care owing to the impact of their wounds, high pressure on hospital outpatient clinics who are unable to fully satisfy the demand, and long waiting times for medical care, among others. There is an urgent need to take measures to solve patients’ difficulties in accessing medical care and to alleviate the burden on their families. The digitally enabled home-based wound care program offers a way to solve these problem; however, such programs are still at the exploratory stage in China and most hospitals do not provide them. It is therefore, necessary to construct an evidence-based implementation DE-HBWC program for chronic wound patients and to explore the effectiveness of its application.

#### Phase II: Evidence-based practice

Evidence-based practice (EBP) refers to methods that combine individual clinical expertise with the best clinical evidence available from systematic studies (24) and patient values to achieve optimal health outcomes. EBP requires nurses to quickly access and evaluate evidence before integrating it into clinical practice (25). Two graduate nursing students were assigned to conduct a literature review in order to critically evaluate and summarize the evidence for development of a DE-HBWC program for patients with chronic wounds.

A comprehensive search strategy was executed across three international databases (PubMed, EMBASE, Web of Science) and three Chinese databases (China National Knowledge Infrastructure [CNKI], Wanfang Database, VIP Database for Chinese Technical Periodicals), covering peer-reviewed articles published between August 2014 and August 2024. The search employed the following Boolean operators and MeSH/Emtree terms: [(home-based) OR (home care services) OR (hospital at home) OR (telehealth*) OR (mHealth*) OR (telecare) OR (eHealth*) OR (telemedicine) OR (digital health*) [AND] (wound care) OR (chronic wounds)]. Filters restricted results to title/abstract fields and journal articles. Additional studies were identified through backward reference tracking (snowball method). Reference tracking was carried out to identify additional potentially relevant references. The inclusion criteria were studies with designs such as randomized controlled trials (RCTs), cohort studies, and quasi-experimental studies, and those published in English or Chinese. The exclusion criteria were non-peer-reviewed sources like conference proceedings, magazines, news articles, theses, dissertations, abstracts, editorials, systematic reviews, as well as gray literature such as technical reports and unpublished data.

#### Phase III: Determinants

This step entails the identification of determinants of implementation, including facilitators and barriers. CFIR is one of the most commonly used theoretical frameworks in implementation science, and comprises a menu of structures related to effective implementation to guide or evaluate the implementation of evidence-based practices. CFIR was first proposed by Damschroder et al. in the United States in 2009 and updated in 2022 as the Consolidated Framework for Implementation Research version 2.0 (CFIR 2.0) (23, 26). The theoretical framework consists of five domains, namely, the innovation domain, outer setting domain, inner setting domain, individuals domain, and implementation process domain. In this study, we applied the CFIR 2.0 framework and combined findings from the literature with clinical practice to identify interview questions for chronic wound patients, patient caregivers, wound specialty nurses, and healthcare facility administrators, addressing internal and external factors. Furthermore, we conducted in-depth interviews with these four groups using a semi-structured interview method. Researchers interviewed 10 chronic wound patients, 10 caregivers, 8 wound specialty nurses, and 5 healthcare facility administrators, each for 30–45 min. Table 1 shows the interview guides. Then, we identified barriers and facilitators to the implementation of the DE-HBWC program in patients with chronic wounds according to the CFIR 2.0 framework.

**Table 1:** Interview guides based on CFIR 2.0 framework.

Domain	Innovation	Outer setting	Inner setting	Individuals	Implementation process
Chronic wound patients					
Questions	How would you describe your experience using the digital tools (e.g., AI wound assessment app) for home-based care? What challenges, if any, did you face while using these tools?	How easy or difficult is it for you to access healthcare resources (e.g., supplies, specialists) in your community? Has your family or social network supported your participation in this program?	How well do you feel your care team (nurses, doctors) communicates with you about your wound care plan?	What motivates you to follow the remote monitoring schedule? Have you ever felt hesitant to use the technology? If so, why?	What suggestions do you have to make this program more patient-friendly?
Patient caregivers					
Questions	How has using digital tools (e.g., telehealth platforms) changed your caregiving routine? What features of the program have been most helpful or frustrating?	Are there community resources (e.g., support groups, transportation) that could better support your caregiving role?	How clearly do healthcare providers explain the care plan to you? Do you feel equipped to handle emergencies (e.g., sudden wound deterioration)?	What concerns do you have about balancing caregiving with other responsibilities?	How could the program better involve caregivers in decision-making?
Wound specialty nurses					
Questions	How does the digitally enabled home-based wound care program fit into your current workflow? What adjustments are needed to improve the usability of digital tools (e.g., AI reporting)?	How do local policies or insurance coverage affect your ability to deliver home-based care?	What organizational resources (e.g., training, staffing) are critical for successful program implementation? Are there communication gaps between your team and primary care providers?	How confident do you feel using telehealth platforms for wound assessments?	What strategies would ensure long-term sustainability of this program?
Healthcare facility administrators					
Questions	How does this program align with your institution’s strategic goals?	What external partnerships (e.g., insurers, tech companies) are needed to scale this program?	What financial or staffing investments are required to maintain the program? How do you measure the program’s success (e.g., cost savings, patient outcomes)?	How do staff attitudes toward technology influence program adoption?	What policy changes would improve the program’s integration into routine care?

#### Phase IV: Action

Action refers to the implementation of a strategy or intervention. This step was based on literature research results and identification of the determining factors for implementation to develop the initial draft of the DE-HBWC intervention plan. An expert meeting is held to revise the intervention plan, and 10 chronic wound patients are recruited for a one-month small-sample pre-experiment to create an applied version of the intervention plan. Participants in the expert meeting included 10 experts specializing in hospital management, wound specialist nursing, wound specialist physicians, information engineering technology and implementation science. Eligibility criteria will require all participants to hold a master’ s degree or higher and possess an associate senior-level professional title or equivalent. Subsequently, a randomized controlled trial of the DE-HBWC intervention plan will be conducted. The main content of the randomized controlled trial is described.

### Recruitment and screening of participants

The participants will be adult patients (aged at least 18 years) with chronic wounds and at least one experience of visiting a wound treatment clinic. To ensure positive and successful registration, we will adopt a proactive recruitment strategy. Participants will be recruited by placing posters at Fujian Provincial Hospital and creating electronic posters that will be shared and promoted by research team members and hospital staff on social media platforms such as WeChat (one of the most widely used chat apps among Chinese citizens). Specialized nurses in wound treatment clinics will actively promoted recruitment of patients who attend for treatment, search for their electronic medical records, and perform other related tasks. Potential participants who respond to the recruitment strategy and contact the researchers will be screened in person or over the phone. The research purpose and process will be carefully explained to participants who met the inclusion and exclusion criteria. Patients who agree to participate will be recruited after signing an informed consent form and completing a general information survey and baseline data survey.

### Inclusion criteria

Patients are eligible for inclusion if they meet all of the following eligibility criteria: (1) age≥18 years; (2) histologic criteria of the Chinese Medical Association for chronic wound (27); (3) the duration of wound was 1 month or more; (4) the patient or an accompanying person has the ability to operate a smartphone; and (5) provision of informed consent and a signed informed consent form.

### Exclusion criteria

Patients who meet any of the following criteria will be excluded: (1) receiving palliative care or having malignant or fungal tumors, wounds with blind-end tracks such as sinus tracts, dry gangrene, exposed blood vessels, and other non-healing wounds; (2) suffering from mental illness or not cooperating with care; (3) suffering from autoimmune diseases or other serious comorbidities; and (4) hospitalized patients.

### Randomization, allocation concealment, and blinding

Before randomization, a researcher not involved in subject recruitment and data collection will prepare a randomization list with 100 sets of numbers, using Research Randomizer software.^1^ The study subjects will be randomly divided into an intervention group and a control group in a 1:1 ratio by personnel who will not directly participate in this study. Then, random numbers will be assigned to the selected groups of research subjects, and they will be sorted from small to large according to the size of the random numbers. The first half will be the control group, and the second half the intervention group. The random numbers will be sealed and handed over to personnel responsible for random allocation for proper safekeeping. They will not participate in the inclusion of the study subjects and subsequent intervention processes. However, because of the nature of this intervention, it would not be not possible to blind the participants and intervention staff, although the data collectors and statisticians in the study will be blinded.

### Sample

The recruitment and screening of all participants were conducted on the online platform of Fujian Provincial Hospital’s WeChat official account or the hospital’s offline wound treatment clinic. This is a randomized controlled trial consisting of two branches and three evaluation stages (T0, baseline; T1, 12-week post intervention; T2, 24-week follow-up). The intervention-group participants received DE-HBWC project wound treatment at their own homes, while the control group participants received treatment at traditional hospital wound-treatment clinics. The required sample size was estimated using PASS v15.0 (NCSS, LLC, United States), based on a completely random design, to compare the mean values of two independent samples. Based on our previous wound treatment results for the healing time of chronic wounds and using two-sample T-tests assuming equal variance procedure of PASS v15.0, μ1 = 38.64, μ2 = 32.54, *σ* take 8.21, a sample size of 40 participants per group was determined to be sufficient to detect an effect with a type 1 error rate of 5 and 90% power. Assuming a drop-out rate of 20%, a total of 100 participants will be needed, with 50 participants in each group.

#### Intervention, DE-HBWC arm

The DE-HBWC program was devised through three phases of crafting the research questions, EBP, identifying the barriers and facilitators to intervention implementation, and, ultimately, through meetings with experts and pre-experiments. The DE-HBWC program is a personalized one-on-one treatment program for chronic wound patients delivered by specialist wound nurses using a combination of online and offline intervention modes. Conducting remote video visits through electronic facilities weekly to evaluate wounds and provide guidance on dressing changes online, and offline home medication change, assessment, and health guidance services are provided by wound specialist nurses every 2 weeks. During weekly remote assessments, all aspects of wound care are carefully assessed by a wound specialist nurse and documented with the assistance of a graduate nursing student. This includes wound morphology, which encompasses tissue type, dimensions, and color profiles, as well as exudate metrics such as volume, consistency, and the presence and severity of odor. The assessment also evaluates the periwound status, focusing on skin integrity, sinus tract characteristics, and signs of infection. Furthermore, pharmacological interventions are thoroughly recorded, including antimicrobial therapies and adjunctive agents, along with detailed application protocols. Lastly, the documentation tool supports intervention tracking by capturing dressing protocols, debridement frequency, and the provision of health education to patients and caregivers.

Nurse home visits are scheduled biweekly, informed by evidence-based guidelines and optimized for resource efficiency. Frequency adjustments follow predefined clinical criteria (e.g., wound progression, infection signs) and are approved by the supervising physician. Remote monitoring data (e.g., wound images) inform visit prioritization, ensuring high-risk patients receive timely in-person care. Wound specialist nurses completed a four-stage competency-based training program, including theoretical instruction, technical skill simulations, protocol-specific workflows, and ongoing supervision. The Nursing Department monitors fidelity by reviewing protocol deviations on a weekly basis. The dressing change is customized by the wound specialist nurse to the individual patient’s situation in regard to the wound. The duration of the intervention was 12 weeks; if the patient’s wound was completely healed before 12 weeks, there was no dressing change—only assessment and guidance; however, if the wound was not healed after 12 weeks, the intervention could be continued according to the patient’s wishes. Patients with chronic wounds are consulted through the developed internet hospital, and data such as basic patient information, questionnaires, and wound pictures are managed using the chronic disease management system already developed by the hospital.

We aim to create a supportive system for patients with chronic wounds. This includes an online community where patients can share their care experiences and feel less isolated. We also provide easy-to-understand educational materials like animated videos and illustrated medication guides, which are sent out weekly through our chronic disease management platform. Additionally, we offer a 24-h nursing hotline. Trained wound nurses will be available to answer urgent questions from families, such as what to do if a wound starts bleeding or gets worse. To ensure participants follow the program guidelines, we use a game-based incentive system. Participants can earn points by uploading wound photos, reading health education materials, completing assessments, or joining activities. These points can be exchanged for nursing supplies or used to book services more quickly. Wound care reminders are customized based on each patient’s daily routine. We also send personalized reminders for wound care based on each patient’s routine.

The program is overseen by a wound care specialist physician who is responsible for reviewing patient progress biweekly through electronic records. This physician also approves any necessary adjustments to treatment plans. Nursing graduate students are responsible for assisting with text recording and data organization. Additionally, nurses play a pivotal role in coordinating care with primary care providers utilizing the hospital’s integrated electronic health record system, ensuring seamless communication and continuity of care. The Deputy Director of Nursing is responsible for overseeing the project implementation and ensuring quality control. Patients are provided with access to a telehealth hotline designed to address urgent concerns, such as sudden onset of pain, fever, or purulent drainage. This resource ensures that patients can promptly seek assistance for complications that may arise during their treatment. The escalation protocol ensures tiered clinical responses: (1) patients with suspected wound deterioration (e.g., increased erythema, purulent drainage, or systemic symptoms) initiate real-time telehealth consultations with certified wound specialist nurses; (2) nurses triage cases to physicians for urgent directives, prioritizing same-day in-person evaluations for high-risk scenarios; and (3) physicians determine hospitalization needs based on standardized severity criteria (e.g., sepsis risk, necrotizing infection).

#### Control, TH arm

Participants in the control group underwent offline traditional treatment at the Wound Treatment Clinic of Fujian Provincial Hospital, and the intervention cycle and dressing change principles were the same as those for the intervention group. Patients in the control group attended the Wound Treatment Clinic every other day for dressing change, assessment, and health guidance. The frequency of dressing changes is flexibly adjusted by the wound specialist nurses according to the patient’s patient wound healing status. Wound specialist nurses delivered the interventions, while nursing graduate students collected patient data using the hospital’ s in-house chronic disease management platform.

### Study outcome measures

The primary outcome measures are wound healing based on the commonly used Bates-Jensen Wound Assessment Tool (BWAT) (28) and the Wound treatment table, which was formulated by our team, based on previous research (29), to evaluate the size, depth, and exudation of chronic wounds; drugs; dressings used for treatment; cost; healing duration; as well as the round-trip transportation and waiting time. Wound images will be acquired weekly and recorded and analyzed using Three Dimensions (3D) AI reconstruction technology. Empirical evidence demonstrates that 3D wound measurement devices achieve comparable accuracy to traditional nurse-performed measurements using manual scales (*p* > 0.05), while significantly reducing procedural time (*p* < 0.05) (30). Caregivers or patients can upload wound photographs through a smartphone application. The AI system automatically generates a 3D model of the wound and identifies areas that require attention, such as those with a “high infection risk.” This tool is particularly beneficial for rural or homebound patients, as it enables specialists to conduct remote assessments of wounds, thereby minimizing the necessity for in-person visits. The secondary outcome measures included participants’ quality of life, health status, wound self-management, satisfaction, and cost-effectiveness. The Chronic Wound Patient Quality of Life Scale (Wound-QoL) (31), Chronic Wound Self-Management Scale (32), and the 36-Item Short Form Survey (SF-36) (33) will be used to evaluate health, quality of life, and wound self-management. Satisfaction survey forms will be used to measure patient satisfaction with wound care. The economic benefits of intervention measures will be evaluated by measuring the cost–benefit and incremental cost–benefit ratio of wound care for the two groups of participants. More information about the sensitivity and specificity of the assessment methods can be found from the reference 28, 29.

All outcome measures and assessment time points are shown in Table 2.

**Table 2:** Assessment outline and timelines.

Measures/time point	Pre-intervention	Intervention period	Post-intervention	Follow-up
	0 weeks		12-week	24-week
Eligibility assessment				
Inclusion criteria	×			
Exclusion criteria	×			
Informed consent	×			
Allocation	×			
Intervention				
DE-HBWC group		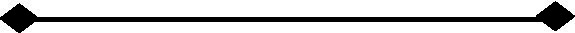		
Traditional hospital treatment control group		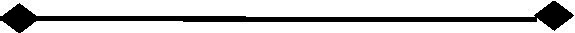		
Demographics and other baseline characteristics	×			
Primary outcomes				
Wound healing (BWAT, Wound treatment table)	×	× (Biweekly)	×	×
Wound healing(3D AI wound image)	×	× (weekly)	×	×
Secondary outcomes				
Quality of life (Wound-QoL)	×		×	×
Self-management ability (WSMS)	×		×	×
Health status (SF-36)	×		×	×
Economic effect (CER, ICER)			×	
Satisfaction (Satisfaction survey form)			×	

×, Time point in the trial at which the assessment took place. ◆, Start or end time point of the trial. BWAT, Bates-Jensen Wound Assessment Tool. 3D, Three Dimensions. AI, artificial intelligence. Wound-QoL, Chronic Wound Patient Quality of Life Scale. WSMS, Chronic Wound Self-Management Scale. SF-36, 36-Item Short Form Survey. CER, cost-effectiveness ratio. ICER, incremental cost-effectiveness ratio.

### Data collection and statistical analysis

Enrolled subjects will be scheduled to complete the baseline data collection. Data will also be collected after 12 and 24 weeks for outcomes, and data on the primary outcome, wound healing, will be collected biweekly. All data analysis will be performed using SPSS Statistics v21.0 (IBM). A two-tailed approach will be used, with *α* level set to 0.05 for testing. To ensure that outcome evaluators and data analysts are not influenced by group assignment, they will not interact with participants, except in the case of outcome evaluators during data collection. Descriptive statistics will be used to summarize demographic and other baseline characteristics. Tests of normality of data distribution were validated using Kolmogorov–Smirnov. After approaching normality for each variable, two-sample independent *t*-tests, x^2^-tests (or Fisher’s exact tests), or nonparametric tests, as appropriate, were used to compare normally distributed variables between the intervention and control groups. If necessary, the results will be adjusted based on potential confounding factors such as age, gender, and education level. Statistical analysis will be based on the intention-to-treat (ITT) method (34).

Generalized linear mixed effects models (GLMM) will be used to estimate the odds ratio (OR) and 95% confidence intervals (CIs) for assessing whether changes in outcome indicators are significantly different over time. Models include randomized groups with time as a fixed effect and time × group as an interaction effect to account for the correlation between repeated observations for each participant. Characteristics such as patient age, gender, wound type, and wound size will be adjusted for as covariates. In addition, to explore the effect of baseline characteristics on intervention effect sizes, we will conduct subgroup analyses by testing for interactions between subgroups and intervention effects. Predefined subgroup analyses will be conducted for baseline characteristics including age, gender, education, monthly income, wound type, and wound size. The main outcomes of the subgroup analyses are *p*-values for the group × time × characteristics interaction coefficients and estimates (95% CIs) of the differences between the intervention and control groups within each subgroup. Finally, sensitivity analyses will be performed by interpolating missing data using a random forest model to improve the reliability of our findings.

### Data management and quality control

Data from medical records and questionnaires will be collected and stored in a secure electronic data capture system, named “the chronic disease management system.” Other raw data files will be securely stored online and securely transferred to the statistics team for analysis. Patient wound images will be transferred to an online server for 3D artificial intelligence algorithms to reconstruct the wound. Files will be linked to the database only through unique participant identifiers.

The data collection system will utilize a rigorously designed logical progression and conditional constraints to improve the accuracy and efficiency of questionnaire completion. Data collectors will undergo two rounds of standardized online training upon onboarding to ensure proficiency in the use of the data collection system and a uniform understanding of the collected item content. All data collectors will take an online exam and only those who pass the exam will be allowed to proceed to the formal data collection phase. The quality control team will scrutinize each entry in the dataset to prevent any incorrect or missing data.

#### Phase V: Long-term

In the last step, based on the results of the above research, a DE-HBWC implementation plan will be developed that can be continuously implemented in the long term to promote application. The long-term implementation plan is anchored in a multi-stakeholder, data-driven approach. Drawing from barriers identified in Phase III and adherence metrics from Phase IV, we will advocate for policy reforms to institutionalize home-based wound care. Continuous quality improvement will be enabled by a digital dashboard tracking healing rates and caregiver burden, with annual audits informing protocol refinements. Sustainability will be ensured through public-private partnerships for technology maintenance and community health worker training programs. During implementation, the plan can be dynamically adjusted as necessary to make it suitable for real-world implementation.

Firstly, we plan to include the DE-HBWC program in the hospital’s yearly goals and try to get it covered by Medicare. We’ll set up a special fund for things like telemonitoring equipment, nurse training, and patient support. Secondly, we’ll create a group of wound care experts from different hospital areas. They can consult and refer tough cases through our platform. We’ll also work with information technology to update the AI algorithms every year. Next, we’ll make a standard operating manual for DE-HBWC. We’ll provide toolkits with protocol templates, training courses, and data collection tools to make it easier to implement. We’ll break down components like training, technology, and policies into modules so that different hospitals can adapt them as needed. Then, we’ll train the first batch of certified wound specialist nurses and create a “DE-HBWC certified nurse” qualification with performance incentives. Lastly, we will set up a patient advisory board and hold feedback meetings every 6 months. We will focus on and address the top three improvement suggestions, such as simplifying the app operation process.

#### Scale: monitoring and evaluation

The implementation of interventions will be scientifically monitored and evaluated in the third to fifth stages of the research design and methods section. Furthermore, after completion of the 12-week DE-HBWC program intervention, we will perform a 12-week randomized controlled trial to evaluate the impact of an “incentive” implementation strategy within the DE-HBWC intervention group. This trial will assess both adherence rates (e.g., completion of remote assessments) and clinical outcomes (e.g., wound healing progression) to determine the strategy’s efficacy in optimizing engagement and therapeutic success. The “incentive” implementation strategy involves providing small financial reimbursements to participants in the intervention group to enhance adherence to telehealth consultations and data submission. This strategy is distinct from the clinical components of the core intervention (e.g., wound care protocols, AI monitoring) and serves solely to address behavioral barriers to participation. Control group participants receive identical clinical care without financial incentives.

#### Oversight and monitoring

The Trial Management Group, consisting of the Principal Investigator, the chief researcher, 1 nursing research staff member, 2 Wound specialist nurse, and 2 nursing master’s students, will meet on a monthly basis to oversee the study. The chief researcher will supervise the daily operation of the experiment and be responsible for the experiment.

#### Assessing adherence and strategies to improve adherence to the intervention

The compliance of patients in the intervention and control groups will be evaluated by measuring the rates of participation in online remote video visits, image acquisition, and offline wound treatment. If the patient does not participate as required, we will contact them via WeChat or phone. The intervention group will be exempt from on-site service fees. Two groups of patients who complete the prescribed number of interventions will have the opportunity to receive a reduction in wound treatment costs.

#### Criteria for discontinuing or modifying allocated interventions

Every participant has the right to withdraw from the trial at any time. Participants will be terminated from the trial if, in the opinion of the investigator, it is necessary to do so for any reason, including ineligibility, adverse events, withdrawal of consent. Participants randomly assigned to the intervention group had the option of participating in traditional hospital wound treatment in the control group if they did not want to continue with the intervention. If a participant withdraws due to a related adverse event, the investigator will schedule a follow-up visit or phone call until the adverse event is resolved or stabilized. Adverse events unrelated to the intervention will be followed up until completion of the trial. Participants who stop the trial will have anonymized data collected until the exit time point. Any assessments not completed will be considered missing data. The reason for withdrawal will be recorded. Participants who withdraw after being randomized will not be replaced. Participants found to be ineligible after randomization will be withdrawn and no further data will be collected thereafter.

#### Patient and public involvement

Patients or the public were not involved in the design, conduct, reporting, or dissemination plans for this study.

## Discussion

This study aimed to evaluate the impact of the DE-HBWC program on wound healing and quality of life in chronic wound patients and to assess its economic benefits. Although DE-HBWC programs have been used in the treatment of chronic wounds and have achieved promising results, some implementation-related problems persist (18, 35). This study is a new exploration of the implementation of a DE-HBWC program based on a scientific framework employed in implementation research, namely, the five-step PEDALs model research paradigm. Barriers to the implementation of the DE-HBWC project as well as facilitators were identified through qualitative interviews. The intervention program was formulated based on the results of the literature research and qualitative interviews, and the program was modified and improved through expert meetings and small sample pre-testing to ensure a more rigorous, scientific, operable, and feasible program development process.

The present DE-HBWC program potentially represents a convenient and low-cost wound care model for chronic wound patients, enabling them enjoy hospital-level care in a home setting. It solves the problem of the inconvenience caused to patients in leaving their homes, having to undertake journeys to hospital, and waiting to receive in-person medical care, and if therefore especially beneficial for patients with mobility problems such as those who are disabled or older adults. In addition, a rigorous methodological design will be used to reduce bias, including randomization, blinding, use of a control group, recruitment strategies, rigorous eligibility screening, and pilot trials. Potential selection bias in the control and intervention groups will be minimized by the above strategies.

### Limitations and future directions

The study was relatively short and conducted at only one hospital. Therefore, the results need to be validated with a larger sample size and through multi-center studies. Collaborate with policymakers to investigate reimbursement models for home-based digital care within the framework of China’s national health insurance system. Additionally, evaluate non-financial incentives, such as gamification and peer support, to improve adherence in resource-limited settings.

## Footnotes

^1^ http://www.randomizer.org/
